# The New Role for an Old Kinase: Protein Kinase CK2 Regulates Metal Ion Transport

**DOI:** 10.3390/ph9040080

**Published:** 2016-12-21

**Authors:** Adam J. Johnson, Ming J. Wu

**Affiliations:** 1 School of Science and Health, Western Sydney University, Locked Bag 1797, Penrith NSW 2751, Australia; a.johnson@westernsydney.edu.au; 2 Molecular Medicine Research Group, School of Medicine, Western Sydney University, Locked Bag 1797, Penrith NSW 2751, Australia

**Keywords:** protein kinase CK2, metal toxicity, genome-wide screen, metal ion transport, zinc channels, therapeutic targets

## Abstract

The pleiotropic serine/threonine protein kinase CK2 was the first kinase discovered. It is renowned for its role in cell proliferation and anti-apoptosis. The complexity of this kinase is well reflected by the findings of past decades in terms of its heterotetrameric structure, subcellular location, constitutive activity and the extensive catalogue of substrates. With the advent of non-biased high-throughput functional genomics such as genome-wide deletion mutant screening, novel aspects of CK2 functionality have been revealed. Our recent discoveries using the model organism *Saccharomyces cerevisiae* and mammalian cells demonstrate that CK2 regulates metal toxicity. Extensive literature search reveals that there are few but elegant works on the role of CK2 in regulating the sodium and zinc channels. As both CK2 and metal ions are key players in cell biology and oncogenesis, understanding the details of CK2’s regulation of metal ion homeostasis has a direct bearing on cancer research. In this review, we aim to garner the recent data and gain insights into the role of CK2 in metal ion transport.

## 1. CK2—A Pleiotropic Kinase

Protein kinase CK2 was first discovered in 1954 [1]. It is one of the earliest kinases in the kinome which currently has about 500 members [1,2,3]. Over the ensuing decades, its structure, function and substrates have been progressively characterized [4,5,6]. Despite the enormous progress in characterisation of its roles in cell proliferation, differentiation and anti-apoptosis, several aspects of CK2 are yet to be fully understood such as its regulatory mechanisms in response to extracellular signals. Also scarcely known is its role in metal ion uptake and toxicity, which is the topic of this review.

CK2 is a ubiquitous, pleiotropic, serine/threonine protein kinase with a wide range of substrates, and has been referred to as the most pleiotropic protein kinase existing in eukaryotic organisms [5,6]. Originally the enzyme was termed casein kinase 2 due to its phosphorylation of casein as the first substrate used to assay enzyme activity, and the numerical designation 2 to denotes its elution from DEAE-cellulose after the enzyme CK1 [1,6,7,8]. However, casein does not appear to be a physiological substrate for CK2 and, therefore, in 1994 it was suggested that the name be changed to protein kinase CK2 to avoid the misnomer confusion [7,8]. CK2 is an unusual protein kinase in several respects. For examples, it is constitutively active and can use both ATP and GTP as the phosphate donor, thus it is different from the other eukaryotic protein kinases [5,7,9,10,11]. The known substrates of this enzyme are expanding to the thousand [5]. It has been suggested that the proteins phosphorylated by CK2 may make up one quarter of the eukaryotic phosphoproteome [5]. The renowned role of CK2 is its regulation of cell proliferation, including the processes of DNA replication, transcription, tRNA and rRNA synthesis, chromatin remodelling and anti-apoptosis [6,12,13]. With its high pleiotropism, it is not surprising that new aspects of CK2 functionality are continually unravelled, such as we and the others demonstrate that CK2 is involved in metal ion transport [14,15].

## 2. Structure and Function of CK2

The mammalian CK2 heterotetramer is a protein kinase composed of two catalytic subunits (α and α’), bound to a central homodimer of regulatory β subunits. The fact that the holoenzymes are formed spontaneously in vitro from the mixture of individual subunits tells us that there is probably a built-in code for such action amongst their primary structures. Notably, the affinity of CK2 α′ for CK2 β is about 12 times lower than that of CK2 α [16], suggesting that the tetramer ααββ of CK2 could be the dominant species. The amino acid sequences of human α and α’ catalytic subunits are 391 and 350 residues long, respectively. The apparent sizes after purification in vitro are smaller than their theoretical molecular masses (45.144 and 41.213 kDa), due to proteolytic cleavage modifications at the C-terminus [17,18]. The β subunit is much smaller (around 25 kDa) [19,20]. The crystallographic structures of the subunits of CK2 and the holoenzyme demonstrate that the catalytic subunits of CK2 contain the typical architecture found in eukaryotic protein kinases [16,21]. Such architecture consists of two domains: a β-sheet based N-terminal domain, and an α-helical C-terminal domain. The active site is located in a cleft between the two domains [16,17,21]. The main difference between the two catalytic subunits in terms of the three-dimensional structure is found in the CK2 β interface region (β4/β5 loop). Unbound CK2 α typically has a closed β4/β5 loop, while CK2 α’ has an open one [16].

A worthwhile notion in the context of this review is that two zinc ions are involved in the holoenzyme. The CK2 β subunit contains a zinc finger that has been shown to be essential for the homodimerisation of the β subunits [22]. The four cysteines (cys^109^, cys^114^, cys^137^ and cys^140^) of CK2 β are in a zinc finger-like arrangement reminiscent of DNA binding proteins [7]. Mutations to cys^109^ and cys^114^ result in disruption of subunit interactions. Each CK2 β monomer consists of an α-helical N-terminal domain and the zinc stabilising area and a C-terminal “tail” [17,23]. The tail crosses the dimer interface and attaches to the other β monomer. This tail segment has been shown to be essential for holoenzyme formation [23]. The holoenzyme complex is shaped like a butterfly, with the catalytic subunits attached to a central dimer of regulatory β subunits [17]. The arrangement is such that both regulatory subunits make contact with each of the catalytic subunits, while neither catalytic subunit contacts the other [17]. The conservation of the active site of unbound CK2 α and the holoenzyme-bound CK2 α supports the idea that CK2 α is catalytically active in isolation and that CK2 β is not an on/off switch as is found in similar kinases such as cyclin-dependent kinase 2 [17,23].

Its extensive list of protein substrates reflects the pleiotropic nature of CK2 functionality, and is structurally due to the acidic consensus sequences (e.g., -**S**XXE/D-, S for serine which is the most common phosphoacceptor) recognised by the kinase [6,24,25]. The multiple tetrameric forms (α2β2, α’2β2, αα’β2) are present in all animals including mammals, amphibians and insects [17,19,26]. Evidence shows that the formation of human CK2 tetramers occurs via the catalytic subunits attaching independently to a stable dimer of the β subunits [17,23]. The free monomeric α and α’ subunits of CK2 are catalytically active as well in the absence of the β subunit and there is evidence that the discrete subunits possess individual functions different to the functions of the tetramer [27,28,29,30,31]. The α and α’ subunits are structurally analogous but are encoded by different genes [7]. The β subunits in tetramers may provide stability, protect α-subunits against denaturing agents or conditions, modulate activity of the enzyme or alter substrate specificity and interactions with inhibitors [7,32]. It has been noted that catalytic activity is increased 5–10 fold for certain substrates by the presence of the β subunit [7,33]. Unlike mammalian CK2, yeast cells possess two distinct regulatory subunits (*CKB1* and *CKB2*), while the catalytic subunits are commonly referred to as *CKA1* and *CKA2*. Yeast CK2 tetrameric holoenzymes have been found to require both *CKB1* and *CKB2* subunits [34].

As the yeast *Saccharomyces cerevisiae* contributes to our understanding of mammalian cell biology, such as cell cycle control [35,36], and the signalling serine/threonine kinase TOR (target of rapamycin) [37,38], it proves to be a useful tool again towards understanding CK2. The genes of CK2 were first deleted in *S. cerevisiae* by homologous recombination. The yeast cells with disruption of either *CKA1* or *CKA2* genes are still viable; however, disruption of both *CKA1* and *CKA2* genes at the same time is lethal [39]. It is therefore clear that under normal growth conditions the catalytic subunits are compensatory. However, several studies imply that under certain environmental conditions individual subunits confer different phenotypes [14,40,41], and, therefore, cannot be compensated by one another. In terms of the regulatory subunits, deletion of *CKB1* or *CKB2* or both does not lead to lethality. However, in mammals such as mice, homozygous knockout of CK2 β is fatal at the embryonic development stage [42]. While the CK2 α′ subunit appears to be essential only for normal spermatogenesis [43], the disruption of the CK2 α gene in mice leads to death in mid-gestation [44]. Taken together, these structural and functional data tell us three basic points: (1) the tetrameric holoenzymes are essential since disruption of CK2 β would abolish formation of the CK2 holoenzyme and leads to lethality; (2) between the two catalytic subunits, CK2 α is more critical than CK2 α’; (3) both CK2 α and CK2 α’ have distinctive functions.

Since CK2 is constitutively active, its activity does not need help from any other kinases. The alternative ways to regulate its activity are by level of expression, subcellular location of the enzyme, and extracellular signals. It is evident in cancers where CK2 is highly over-expressed [6,45]. Spatiotemporal dynamics of CK2 in the nucleus and cytoplasm are shown in live cell fluorescence imaging [46]. The remaining question is what triggers up-regulation of CK2 expression or changes its nucleocytoplasmic distribution. Heretofore, there are scant details in terms of what regulates dynamic distribution of CK2. A recent study by Kalathur et al. [47] strongly demonstrates that the transcription factor, STAT3 (Signal Transducer and Activator of Transcription 3), regulates CK2 transcription and the protein level in mammalian cells. STAT3 itself is phosphorylated in response to growth factors or cytokines. The up-regulation of CK2 results in phosphorylation of the tumor suppressor, PML (Promyelocytic Leukemia protein), which in turn leads to PML ubiquitination and degradation. As a result, oncogenesis ensues.

Moreover, its activity can be increased and decreased by certain compounds. Under certain conditions, polyamines are known to increase the activity of CK2 [7]. This activation requires a specific concentration of the polyamine and, therefore, may only occur in certain cells, e.g., the dividing cells due to their increased polyamine concentration [7]. On the other hand, polyanionic compounds such as heparin are inhibitory to CK2. It is therefore possible that CK2 activity in the liver is subject to heparin concentration [7,48]. This suggests that CK2 activity is regulated in specific cells and tissues by activating inhibitory compounds.

In vitro assays have demonstrated that divalent metal ions such as Mg^2+^, Mn^2+^ and Co^2+^ are required for CK2 activity, but beyond their optimal concentration these metals are actually inhibitory to CK2 [49,50]. These studies were performed using a substrate that precipitates, such as casein, in the presence of metals such as Mg^2+^ and the inhibition of CK2 in the presence of Mg^2+^ concentrations greater than its optimum is due to casein precipitation [51]. The optimal concentration of Mg^2+^ may represent the point at which Mg^2+^-ATP (required for activity) is highest before precipitation occurs [51]. While this is the case for Mg^2+^, substrate precipitation in the presence of Co^2+^ and Mn^2+^ does not occur and, therefore, the inhibition of enzyme activity at levels above the optimum of these ions may be a regulatory mechanism [51]. Interestingly, when activity is assayed using Mn^2+^ and Co^2+^ instead of Mg^2+^, the preferred phosphoryl donor is GTP rather than ATP [49]. Zn^2+^ is inhibitory to CK2 at concentrations above 150 µM [49]. The inhibition of CK2 by zinc, as well as the reported inhibition of activity found when Ni^2+^ is present, is thought to be via direct interaction with the enzyme, perhaps in a manner similar to Mn^2+^ and Co^2+^ [49,51]. Given that the ionic strength of solution greatly impacts enzyme activity [51] and the requirements of CK2 for zinc in order for functional tetramers to form, there might be certain inextricable relationships between CK2 and metal ions.

## 3. Functional Genomics and Discovery of Novel CK2 Functionality

The yeast *S*. *cerevisiae* is a pioneering organism in functional genomics and systems biology [52]. Since the publication of its genomic sequence [53], complete collections of yeast gene deletion mutants such as the collection from EUROSCARF have become available for functional annotation of individual genes by genome-wide screening. Such an approach acquires the phenotype of a gene deletion mutant observed under a given condition. Based upon the phenotype, the function of that gene can be revealed. Genome-wide screening of deletion mutants has been applied to nickel [54], cadmium [55], arsenite [56,57], lead [58], aluminium [14,59] and chromium [40]. Significantly, the findings from the yeast system are relevant to human beings due to the genomic homology between the two organisms. They share thousands of orthologous genes, accounting for about one-third of the yeast genome [60,61]. Additionally, there exists a high level of conservation between the cellular processes of yeast and those of mammalian cells [62,63]. By means of *S*. *cerevisiae* genome-wide deletion mutant screening, we firstly uncovered that deletion of *CKA2* (CKα’) leads to resistance to Al^3+^ toxicity [14]. Further, the regulatory subunits (*CKB1* and *CKB2*) were shown to be involved in regulating the toxicity of As^3+^ [56]. Significantly, the role of CK2 in regulating metal toxicity is confirmed in neuronal cells [64]. Intriguingly, CK2 regulates both Zn^2+^ and Ca^2+^ [64]. Considering that CK2 is a key player in carcinogenesis and that dysregulation of Zn^2+^ is observed in cancers such as breast, prostate, pancreatic, ovarian and hepatocellular cancers [65,66,67,68,69], this discovery has significant bearing on cancer research.

## 4. CK2 and Metal Ion Transport

The ability to transport ions into and out of the cell is essential for life. Herein, we define ion transport as the process of uptake, sequestration into or release from subcellular organelles, and efflux. Approximately 91 of 118 elements in the periodic table are metals or metalloids, many of which are essential to biological functions, whilst some are toxic. Essential metal ions are required for a range of cellular functions, for example, iron is a cofactor for several redox-active metalloenzymes and zinc is required for maintaining protein structures such as in CK2 and the catalytic activity of thousands of enzymes [70,71]. The metal ion uptake, storage and secretion is tightly controlled, and aberrations in this control can lead to cell death and diseases [72,73].

The compendium of recent studies, including the ones of our laboratory aforementioned, demonstrates that CK2 is involved in metal toxicity and transport [64]. We have shown that deletion of *CKA1*, *CKB1* and *CKB2* result in lower accumulation of intracellular chromium, while deletion of *CKA2* leads to higher accumulation than the wild type [40]. We then screened all four deletion mutants of CK2 (*cka1*∆, *cka2*∆, *ckb1*∆ and *ckb2*∆) against Al^3+^, Zn^2+^, Co^2+^, Cr^6+^, As^3+^ and Cd^2+^, and found that individual subunits confer distinct profiles for metal resistance (unpublished data). The findings are two-fold. They demonstrate that CK2 is indeed involved in metal uptake and toxicity, and that individual CK2 subunits have specific roles such as *CKA2* against Al^3+^, and *CKB1* or *CKB2* against As^3+^ and Cr^6+^. The finding that deletion of CK2 subunits results in metal resistance is supported by the dataset obtained by a different high-throughput profiling approach—transcriptomics. Jin et al. [74] revealed, via transcriptomics of *S. cerevisiae*, that the expression of genes encoding subunits of protein kinase CK2 (*CKB2*, *CKA1*, *CKA2*) was repressed by transitional metal ions, suggesting that CK2 gene expression is undesirable for the cells under metal ion exposure. On the other hand, analysis of the ionomic data generated in a genome-wide yeast screen using overexpression strains indicates that the overexpression of CK2 subunits resulted in an increase of certain metals inside the cell including copper, iron and zinc [75]. Apart from the yeast model organism, a similar finding was demonstrated in mammalian cells [76], in which CK2 transcripts were markedly reduced upon chromium exposure. In a study using mouse epidermal JB6 cells, the phosphorylation of p53 (resulting in p53 DNA binding) by CK2 was found to be reduced in the presence of arsenic [77].

How does CK2 regulate metal ion transport, biochemically? Two studies so far can provide us with some insight. In response to the extracellular stimuli, CK2 was found to phosphorylate the zinc channel, ZIP7 (ZIP is an abbreviation of ZRT, IRT-like Protein), located in the membrane of the endoplasmic reticulum (ER) [15]. Consequently, Zn^2+^ ions in ER stores were released, and cytosolic concentration of Zn^2+^ increased, triggering a cascade of signalling pathways, including the activation of receptor tyrosine kinase and the phosphorylation of AKT and extracellular signal-regulated kinases 1 and 2 (ERK1/2). The end result of such action is enhanced cell proliferation. This finding offers mechanistic explanation, if only partially, to the effect of CK2 on promoting cell proliferation as mentioned previously.

CK2 is also found to regulate epithelial Na^+^ channel activity [78]. The Na^+^ channel is a trimeric protein, composed of α, β and γ subunits. The phosphorylation sites for CK2 are located in the C terminus of β (β_S631_) and γ subunits (γ_T599_). The channel’s activity was inhibited dose-dependently by the selective CK2 inhibitor 4,5,6,7-tetrabromobenzotriazole (TBB). Furthermore, the phosphorylation of the channel by CK2 antagonises the inhibition of Nedd4-2, the E3-ubiquitin ligase, which causes channel ubiquitination and degradation. Intriguingly, CK2 was translocated to the cell membrane upon expression of the wild type Na^+^ channel, but not of the mutant channel lacking both of the phosphorylation sites. This notion sheds light on the topic of CK2 distribution as mentioned earlier. There is likely a pulling force or an attraction between CK2 and its substrate, and evidence suggests that this attraction is structurally due to basic residues in key positions of CK2 recognising the acidic determinants in the substrate for phosphorylation. It is expected that more investigations will be carried out on this front.

Another elegant study conducted on CFTR (cystic fibrosis transmembrane conductance regulator) provides more details on the mode of CK2 action [79,80]. Although CFTR is essentially a chloride channel, much can be learned from its interaction with CK2. Inhibition of CK2 closes CFTR wild type but not the cystic fibrosis mutant channel ∆F508-CFTR [81]. The deletion of phenylalanine (F) of the 508th residue in CFTR abolishes the interaction of CK2 with ∆F508-CFTR, suggesting that phenylalanine residue serves as a docking site in the wild type for CK2 action. Furthermore, ∆F508-CFTR mutant is often degraded before reaching the plasma membrane. As for the membrane-bound mutants, they are unstable. Application of the proteostasis regulator cysteamine and the CK2 inhibitor, epigallocatechin gallate (EGCG) or CX-4945, can reduce the degradation of ∆F508-CFTR, resulting in more mutant channels residing in the membrane, hence alleviation of the symptom of cystic fibrosis patients [79]. Such a study serves as an example for future investigations, which could be relevant to the basic understanding of and therapeutic development for many human disorders involving CK2.

Additionally, to understand the cell’s regulation of metal ions, we must differentiate the essential ions, such as iron and zinc, from the toxic ones, like arsenic and aluminium. In the evolutionary sense, the cell has become accustomed to the essential ions and has built-in mechanisms to maintain their homeostasis for growth and survival. The cell’s response to the toxic metal ions is basically a detoxification process using the cell’s defence mechanisms. The data discussed previously clearly show that CK2 is involved in both categories of the metals. The key question is what senses the intracellular level of a particular ion. There is no certain answer to this question thus far. However, it has been shown that in the presence of increased metal ions, the transcription factor MTF-1 (metal transcription factor 1) is phosphorylated by CK2. Upon phosphorylation by CK2, MTF-1 activates metal responsive genes such as metallothioneins [82]. Metallothioneins are a class of cysteine-rich, metal binding proteins that are thought to play a role in essential metal ion homeostasis and detoxification of toxic metal ions [82].

The evidence for CK2’s role in metal homeostasis is emerging. As previously mentioned, the work by Taylor et al. [15] demonstrates a role for CK2 in zinc homeostasis through regulation of the ER zinc channel ZIP7. There are 14 ZIP channels responsible for zinc uptake. There also exist 10 ZnT channels responsible for zinc efflux from the cytosol. Given that CK2 is found to directly phosphorylate ZIP7, it is likely that it also phosphorylates other zinc transporters. Table 1 shows the various zinc channels and the possible residues that could be phosphorylated by CK2.

**Figure 1:**
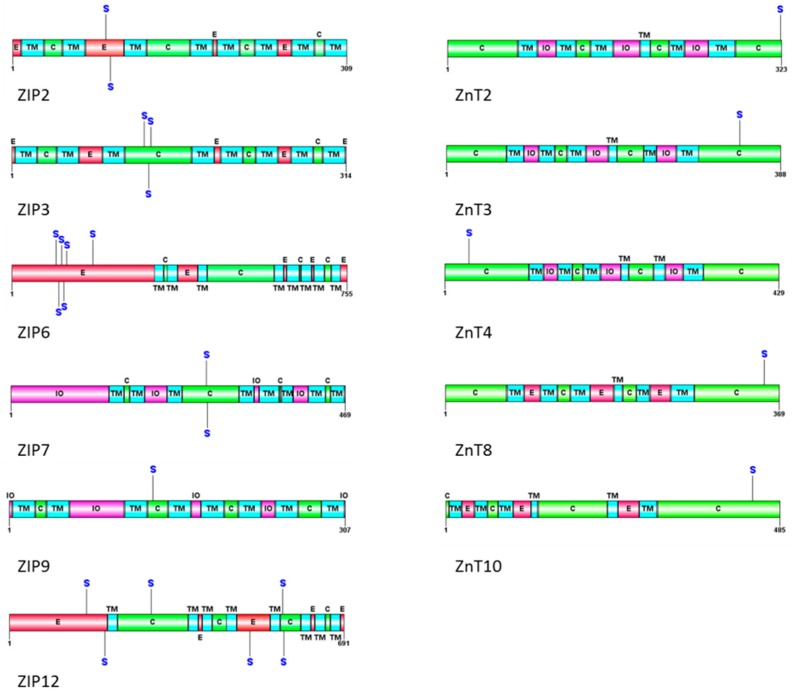
Schematic view of the phosphorylation sites in ZIP and ZnT channels predicted to be phosphorylated by CK2. CK2 phosphorylation sites were predicted using GPS 3.0 software. Transmembrane domains are identified according to Uniprot (http://www.uniprot.org) and the original publications for ZIP2 [84], ZIP3 [84], ZIP6 [84,85], ZIP7 [84,85], ZIP9, ZIP12 [86]. The six TM domains of ZnT2, 3, 4, 8 and 10 are based on an atomic-resolution structure of YiiP [87,88]. TM denotes transmembrane (blue), E for extracellular (red), C for cytoplasmic (green), IO for intra-organellar, S for serine.

Further from Table 1, a schematic view of the predicted phosphorylation sites in zinc channels is shown in Figure 1. It visualizes that many of the ZIP channels (influx to cytosol) and ZnT channels (efflux from cytosol) can be potentially phosphorylated by CK2. While some of these sites are likely not regulatory in nature (due to their extracellular location), some may indeed regulate the activity of the transporter as is seen in the case of ZIP7 [15].

## 5. Metal Transporters Regulated by CK2 Are Potential Therapeutic Targets

In the process of developing therapeutic agents, one of the first steps is to identify a suitable molecular target. For successful precedents, one need look no further than the case of calcium channels. Voltage-gated calcium channels regulate abundant biological functions across various systems and tissues, and numerous drugs have been developed to manipulate calcium channels for treating heart diseases [89,90,91]. Zinc channels and their regulators are also emerging targets.

Zinc is reported to have proliferative and anti-apoptotic properties [92], whilst some reports show that zinc can also be pro-apoptotic [93]. It is becoming clear that the effects of zinc are concentration and tissue specific. It is for these reasons that the role of zinc in cancer is a somewhat enigmatic one. As previously mentioned, zinc levels vary in different cancers. It has been shown that the zinc level is lower in cancers such as ovarian [67], prostate [94] and hepatocellular cancers [69]. This reduction of zinc has, in the case of prostate and hepatocellular cancers, been linked to altered expression of ZIP channels [95]. Intriguingly, zinc was found to induce apoptosis in these three malignancies [96,97,98].

In contrast to the above-mentioned cancers, the level of zinc was shown to be markedly increased in cancers such as breast [99,100] and pancreatic cancers [68]. In both cases, the abnormal zinc level was linked to ZIP transporters and this increased zinc level was found to contribute to cancer progression [68,101,102,103,104]. In the case of pancreatic cancer, depletion of zinc was found to cause apoptosis [105,106].

While these studies demonstrate contrasting roles for zinc in cancerous tissues, they all point to the fact that zinc channels or the mechanisms that regulate them are potential therapeutic targets for a variety of cancers. We have shown that CK2 is involved in the homeostasis and toxicity of zinc in mouse neuroblastoma cells by a mechanism somewhat similar to *S*. *cerevisiae* [64], indicating evolutionary conservation. Distribution and availability of zinc is linked to CK2, not only through our studies, but through the work of others [15,82]. Therefore, not just for its roles in cell proliferation but also as a regulator of metal ion homeostasis, CK2 itself should be explored for development of therapeutic agents.

## 6. Future Perspective

The functional pleiotropism of protein kinase CK2 accentuates the notion that CK2 is at the centre of cellular concatenation, the intricacy of which is still open for delineation. Many questions exist, such as how CK2 regulates metal toxicity. Identification of CK2 substrates, in the context of metal exposure, should be a worthwhile undertaking for future research and drug development. The distinctive effect of CK2 subunits on metal toxicity may serve as a useful tool in understanding the structure and function of the enzyme. We envisage that the current understanding of the role of CK2 in metal transport is merely a prelude to major discoveries in times to come.

## Figures and Tables

**Table 1:** Phosphorylated sites predicted in the zinc channels.

Protein	Site	Exemplar Sequence	Score	Location of Phosphorylation Site
ZIP1	None	-	-	-
ZIP2	S87	MVQNRSASERNSSGD	10.179	Extracellular
ZIP2	S91	RSASERNSSGDADSA	15.628	Extracellular
ZIP3	S125	LETFNAGSDVGSDSE	10.216	Cytoplasmic
ZIP3	S129	NAGSDVGSDSEYESP	16.602	Cytoplasmic
ZIP3	S131	GSDVGSDSEYESPFM	12.602	Cytoplasmic
ZIP4	None	-	-	-
ZIP5	None	-	-	-
ZIP6	S100	HHDHDHHSDHEHHSD	11.864	Extracellular
ZIP6	S106	HSDHEHHSDHERHSD	12.672	Extracellular
ZIP6	S112	HSDHERHSDHEHHSE	12.662	Extracellular
ZIP6	S118	HSDHEHHSEHEHHSD	12.807	Extracellular
ZIP6	S124	HSEHEHHSDHDHHSH	12.309	Extracellular
ZIP6	S183	RNVKDSVSASEVTST	10.324	Extracellular
ZIP7	S275	RSTKEKQSSEEEEKE	16.548	Cytoplasmic
ZIP7	S276	RSTKEKQSSEEEEKE	16.548	Cytoplasmic
ZIP8	None	-	-	-
ZIP9	S132	IGNSHVHSTDDPEAA	11.066	Cytoplasmic
ZIP10	None	-	-	-
ZIP11	None	-	-	-
ZIP12	S160	DEDSSFLSQNETEDI	10.412	Extracellular
ZIP12	S197	KKSGIVSSEGANEST	10.888	Extracellular
ZIP12	S293	QDYSNFSSSMEKESE	11.826	Cytoplasmic
ZIP12	S497	LALNSELSDQAGRGK	9.983	Extracellular
ZIP12	S565	AIGAAFSSSSESGVT	10.276	Cytoplasmic
ZIP12	S567	GAAFSSSSESGVTTT	10.409	Cytoplasmic
ZIP13	None	-	-	-
ZIP14	None	-	-	-
ZNT1	None	-	-	-
ZNT2	S322	CQACQGPSD	10.147	Cytoplasmic
ZNT3	S341	SAHLAIDSTADPEAV	9.921	Cytoplasmic
ZNT4	S32	DTSAFDFSDEAGDEG	13.229	Cytoplasmic
ZNT5	None	-	-	-
ZNT6	None	-	-	-
ZNT7	None	-	-	-
ZNT8	S353	SLTIQMESPVDQDPD	10.017	Cytoplasmic
ZNT9	None	-	-	-
ZNT10	S446	TYGSDGLSRRDAREV	11.562	Cytoplasmic

Note: The protein sequence of each zinc channel was analysed for phosphorylation by CK2 using GPS3.0 software [83], using the high threshold option (reported false positive rate of <2%). This threshold correlates with a cut-off value of 9.84. The table shows the predicted residue position, the sequence, the score (higher score means more likely to be phosphorylated) and the location of the predicted phosphorylation sites.
